# Atypical Value-Driven Selective Attention in Young Children With Autism Spectrum Disorder

**DOI:** 10.1001/jamanetworkopen.2020.4928

**Published:** 2020-05-06

**Authors:** Quan Wang, Joseph Chang, Katarzyna Chawarska

**Affiliations:** 1 Child Study Center, Yale School of Medicine, New Haven, Connecticut; 2 Key Laboratory of Spectral Imaging Technology, Key Laboratory of Biomedical Spectroscopy of Xi’an, Xi’an Institute of Optics and Precision Mechanics of Chinese Academy of Sciences; 3 Department of Statistics and Data Science, Yale University, New Haven, Connecticut

## Abstract

**Importance:**

Enhanced selective attention toward nonsocial objects and impaired attention to social stimuli constitute key clinical features of autism spectrum disorder (ASD). Yet, the mechanisms associated with atypical selective attention in ASD are poorly understood, which limits the development of more effective interventions. In typically developing individuals, selective attention to social and nonsocial stimuli is associated with the informational value of the stimuli, which is typically learned over the course of repeated interactions with the stimuli.

**Objective:**

To examine value learning (VL) of social and nonsocial stimuli and its association with selective attention in preschoolers with and without ASD.

**Design, Setting, and Participants:**

This case-control study compared children with ASD vs children with developmental delay (DD) and children with typical development (TD) recruited between March 3, 2017, and June 13, 2018, at a university-based research laboratory. Participants were preschoolers with ASD, DD, or TD.

**Main Outcomes and Measures:**

Procedure consisted of an eye-tracking gaze-contingent VL task involving social (faces) and nonsocial (fractals) stimuli and consisting of baseline, training, and choice test phases. Outcome measures were preferential attention to stimuli reinforced (high value) vs not reinforced (low value) during training. The hypotheses were stated before data collection.

**Results:**

Included were 115 preschoolers with ASD (n = 48; mean [SD] age, 38.30 [15.55] months; 37 [77%] boys), DD (n = 31; mean [SD] age, 45.73 [19.49] months; 19 [61%] boys), or TD (n = 36; mean [SD] age, 36.53 [12.39] months; 22 [61%] boys). The groups did not differ in sex distribution; participants with ASD or TD had similar chronological age; and participants with ASD or DD had similar verbal IQ and nonverbal IQ. After training, the ASD group showed preference for the high-value nonsocial stimuli (mean proportion, 0.61 [95% CI, 0.56-0.65]; *P* < .001) but not for the high-value social stimuli (mean proportion, 0.51 [95% CI, 0.46-0.56]; *P* = .58). In contrast, the DD and TD groups demonstrated preference for the high-value social stimuli (DD mean proportion, 0.59 [95% CI, 0.54-0.64]; *P* = .001 and TD mean proportion, 0.57 [95% CI, 0.53-0.61]; *P* = .002) but not for the high-value nonsocial stimuli (DD mean proportion, 0.52 [95% CI, 0.44-0.59]; *P* = .64 and TD mean proportion, 0.50 [95% CI, 0.44-0.57]; *P* = .91). Controlling for age and nonverbal IQ, autism severity was positively correlated with enhanced learning in the nonsocial domain (*r* = 0.22; *P* = .03) and with poorer learning in the social domain (*r* = −0.26; *P* = .01).

**Conclusions and Relevance:**

Increased attention to objects in preschoolers with ASD may be associated with enhanced VL in the nonsocial domain. When paired with poor VL in the social domain, enhanced value-driven attention to objects may play a formative role in the emergence of autism symptoms by altering attentional priorities and thus learning opportunities in affected children.

## Key Points

**Question:**

Is there an association between value learning and atypical selective attention in young children with autism spectrum disorder?

**Findings:**

This case-control study included 115 preschoolers with autism spectrum disorder, developmental delay, or typical development whose selective attention was examined after a value learning protocol in social and nonsocial domains. Unlike comparison groups, children with autism spectrum disorder showed strong evidence for value learning in the nonsocial domain but not in the social domain.

**Meaning:**

Atypical selective attention in autism spectrum disorder is associated with enhanced value learning in the nonsocial domain and with poor value learning in the social domain, suggesting that value learning may represent a novel treatment target in autism spectrum disorder.

## Teaser

This case-control study examines value learning of social and nonsocial stimuli and its association with selective attention in preschoolers with and without autism spectrum disorder.

## Introduction

Impaired selective visual attention to social targets, such as the faces of interactive partners, constitutes a hallmark feature of autism spectrum disorder (ASD)^1^ in young children, which has been documented in both laboratory and real-world contexts.^2,3,4,5,6,7,8^ Given the wealth of information conveyed by faces and facial gestures, poor attentional attunement to this class of stimuli during prodromal^9,10,11,12,13^ and early syndromal stages of the disorder^3,8,14,15^ is associated with less favorable social, adaptive, and language outcomes.^16,17,18^ Poor selective attention to social targets is typically accompanied by enhanced attention to certain classes of nonsocial stimuli (eg, geometric patterns or idiosyncratic objects), which may draw the child’s limited processing resources toward less socially relevant aspects of the environment.^2,14,19^ Despite the importance of atypical selective attention in ASD, both as a core feature and a treatment target, its underlying mechanisms remain poorly understood.

In the real world, the number of stimuli placing demand on the sensory system vastly exceeds the processing capacity of an individual. Visual selective attention serves to resolve this problem by prioritizing certain inputs over others for further processing.^20^ A key mechanism driving selective attention in human and nonhuman primates is the appraisal of stimuli values.^21,22,23,24^ The value of a visual stimulus, whether it be social or nonsocial, is defined by the usefulness or relevance of the information it provides to the individual.^23,25,26,27,28^ This information helps individuals gauge the likelihood of favorable or unfavorable outcomes and guides their subsequent behavior. The association between value learning (VL) systems and visual attention have been demonstrated in primates on neuroanatomical and neurophysiological levels,^26,29^ and there is extensive evidence that previously reinforced (high value [HV]) stimuli are detected faster and attended to longer than nonreinforced (low value [LV]) stimuli in typically developing children and adults.^30,31,32,33^ The present study sought to investigate whether observed impairments in selective attention among young children with ASD are associated with atypical VL across social and nonsocial domains.

The VL system constitutes a key component of the brain’s reward system.^14,25^ Although hedonic and motivational components of the reward system have been implicated in ASD,^34,35,36^ few studies have investigated either the process through which initially neutral stimuli acquire reward value for individuals with ASD or the VL component of the reward system. The studies that focus on VL typically examine whether the ability of individuals with ASD to learn the values of nonsocial stimuli (eg, abstract images) depends on reward type, including social (eg, verbal praise or a smiling face) or nonsocial (eg, monetary), using probabilistic reinforcement learning tasks.^37,38^ Adults with ASD perform worse at VL of nonsocial stimuli when the rewards are social, suggesting that social rewards may have a lower motivational currency in ASD^38^; however, developmental data on this topic are lacking. Moreover, although the pairing of nonsocial stimuli with social or nonsocial rewards helps to discern whether rewards have a similar association with learning in individuals with ASD and comparison groups, such a design is not informative about the processes through which initially neutral social and nonsocial stimuli acquire reinforcement value in ASD and whether the purported atypicalities in VL of these stimuli relate to atypical selective attention. One exception is a study^39^ that examined a facet of VL in a small sample of preschoolers with ASD and children with typical development (TD) using a reversal learning task that involved tracking values of stimuli as they changed over time. Compared with the TD group, preschoolers with ASD were less skilled at tracking the rapidly changing values of social stimuli (faces), but the interpretation of observed impairments was complicated by the nature of the reversal task, which captured both VL and value reversal. Although a slight advantage in VL in the nonsocial domain in the ASD group was noted, the study may have been underpowered to detect the association. Moreover, it was unclear if observed deficits are ASD specific or owing to cognitive delays.

To advance our understanding of the mechanisms underlying atypical attentional selection in ASD, we evaluated VL of social (faces) and nonsocial (fractals) stimuli in preschoolers with ASD, TD, or developmental delay (DD) using a novel eye-tracking gaze-contingent task. Because of their high complexity and reliance on verbal instructions, the standard probabilistic learning tasks^37,38^ were not applicable to a study in young and often cognitively impaired participants. Therefore, based on previous evidence in primates^26^ and prior work on VL in young children with ASD,^39^ we developed a novel implicit VL task that requires no verbal instructions and relies on eye movements as the response modality. In the present study, a stimulus was considered to have high informational value if it behaved contingently when fixated on, revealing either positive affect (social condition) or an attractive visual display (nonsocial condition). Compared with prior work,^39^ the task was focused on VL alone (without the reversal learning component), contained more training trials, and used a more stringent test of VL.

Herein, it was investigated whether VL was effective in enhancing selective attention to previously reinforced faces and fractals and whether one stimulus domain was more (or less) altered by the VL in young children with ASD compared with DD and TD groups. If social VL is selectively impaired in ASD, we would expect the ASD group to perform worse in the face condition than the DD and TD groups. If nonsocial VL is enhanced in ASD, we would expect the ASD group to outperform the DD and TD groups in the fractal condition. If VL is impaired across social and nonsocial stimuli in ASD, we would expect poorer performance in both face and fractal conditions in the ASD group compared with the DD and TD groups. We also examined if VL in social and nonsocial domains was associated with the severity of autism symptoms.

## Methods

### Study Participants

This case-control study was approved by the Human Investigation Committee of the Yale School of Medicine, and written informed consent was obtained from all parents before testing. The initial group of participants included children with ASD (n = 55; mean [SD] age, 38.55 [15.40] months), DD (n = 35; mean [SD] age, 44.99 [18.99] months), or TD (n = 38; mean [SD] age, 36.43 [12.07] months) recruited between March 3, 2017, and June 13, 2018, at a university-based research laboratory. Participants with ASD or DD were recruited from consecutive referrals to the Yale Toddler Developmental Disabilities Clinic. Participants with TD were recruited through online advertisements. Diagnoses were assigned by a team of expert clinicians. Details are available in the eMethods in the Supplement. This study followed the Strengthening the Reporting of Observational Studies in Epidemiology (STROBE) reporting guideline.

### Apparatus and Stimuli

**Figure 1.**
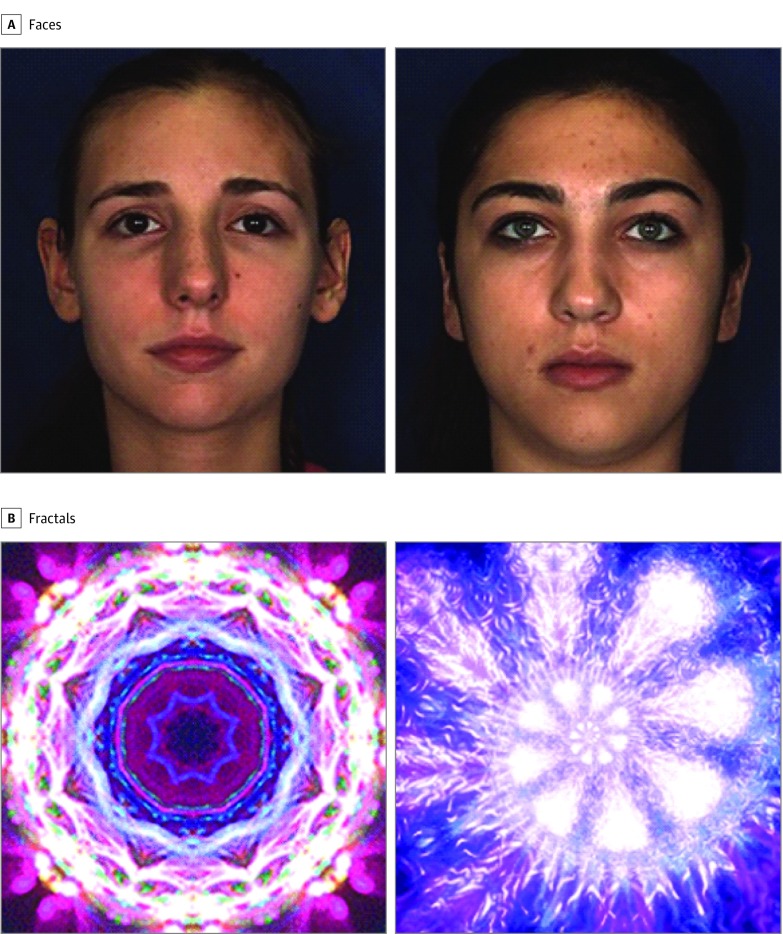
Static Representation of the 2 Types of Stimuli Used in the Experiment (Faces and Fractals) A and B, Facial stimuli were obtained from the Dynamic Facial Database BU-4DFE 3D.^41^ Fractal stimuli were produced using a fractal generation software program based on a generic algorithm.^42^

The gaze-contingent VL task was implemented on an SR EyeLink 1000 Plus 500 Hz (SR Research Ltd) eye tracker configured for remote tracking of gaze behavior using a 5-point calibration procedure. Eye-tracking data were processed using custom software written in MATLAB (The MathWorks, Inc), which incorporated standard techniques for processing of eye-tracking data, including data calibration, online contingent event recording, blink detection, and region-of-interest analysis.^40^ A single pair of faces and a single pair of fractals were presented as stimuli, and all participants were exposed to these same 4 stimuli (Figure 1).^41,42^ The perceptual saliences of the face and fractal stimuli were equated across static and dynamic exemplars within each category. Details are available in the eMethods in the Supplement.

### Procedure

**Figure 2.**
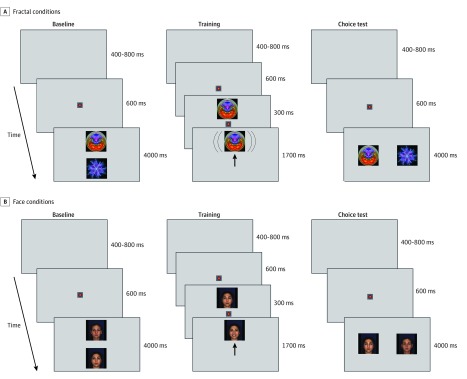
Schematic Representation of a Single Trial of the Value Learning Task in the Face and Fractal Conditions A and B, In the baseline trials, a pair of static stimuli (faces in the face condition and fractals in the fractal condition) is presented in 2 of 4 randomly selected locations. Baseline consists of 4 trials. Each of the baseline trials began with a presentation of a central fixation point for 600 milliseconds (ms). The offset of the central fixation point coincided with the presentation onset of the pair of stimuli (faces or fractals) lasting for 4000 ms. A gray screen was presented during each intertrial interval lasting randomly between 400 and 800 ms. In the training trials, during a high-value (HV) trial (shown), the fixation (marked as an upright arrow) on an HV object results in the activation of a dynamic display (smiling face or revolving fractal). During a low-value (LV) object trial (not shown), fixation on the LV object results in no change to its display. Training consists of 48 trials. Each trial started with the presentation of a central fixation point (multicolor pulsating circle) for 600 ms, followed by the presentation of either the HV or LV stimulus in 1 of 4 randomly selected locations for 2000 ms. There was a 300-ms overlap between the presentation of the central fixation point and the stimulus to diminish the likelihood that participants would look away from the screen before the onset of the peripheral stimulus. In the choice test trials, a pair of static stimuli is shown in 2 of 4 randomly selected locations. Choice test consists of 6 trials, where locations of the HV and LV objects are randomized, with the same structure as the baseline trials.

Each participant was exposed to 2 conditions of the VL task, namely, social (face) and nonsocial (fractal). The procedures for each condition consisted of baseline, training, and choice test phases (Figure 2). In each condition, the stimuli and rewards were from the same category: orienting gaze toward one of the faces in the pair (hereafter called the HV face) activated a smiling sequence in the face, whereas orienting gaze toward the other face in the pair (hereafter called the LV face) elicited no change to the display. The same principle applied to pairs of fractal stimuli, whereby HV fractals revolved slowly but LV fractals remained stationary. No verbal instructions were given to the participants. Each baseline phase consisted of 4 trials; during each trial, the HV and LV static stimuli (eg, 2 faces) were presented side by side for 4 seconds in 2 of 4 possible locations, selected at random. The training phase consisted of 48 trials during which the HV and LV stimuli were presented 24 times each in random order, and stimuli were never presented in the same location for more than 2 consecutive trials. If a child fixated on an HV stimulus during training, it underwent a visual transformation: a fractal would revolve, and a face would smile. However, if the child failed to fixate on the HV stimulus, it remained static. If a child fixated on an LV stimulus, the stimulus remained static. The choice test phase consisted of 6 trials with the same structure as the baseline trials, and its purpose was to establish whether the training trials altered selective attention to HV and LV stimuli. The order of presentation was randomized across participants so that in the ASD group 25 of 48 (52%) completed the face condition first compared with 17 of 31 (55%) in the DD group and 19 of 36 (53%) in the TD group (*P* = .97). Details are available in the eMethods in the Supplement.

### Outcome Measures

As a preliminary analysis, a mean dwell time was computed for each individual across both test phases (baseline and choice test), conditions (faces and fractals), and stimulus values (HV and LV), averaged across trials. The mean dwell time at baseline was used to examine preexisting attentional biases toward social vs nonsocial stimuli. Next, to evaluate if there were any ASD-specific impairments in the disengagement of attention or the generation of reactive saccades during training, saccadic reaction time (SRT) indexes were calculated by averaging the intervals between the onset of the peripheral stimulus and the time when the participant fixated on the stimulus. Finally, dwell times were used to compute HV preference proportions, defined as the mean dwell time on the HV stimulus divided by the total mean dwell time on both HV and LV stimuli. The HV preference proportion at baseline was examined as a check on the randomizations of the reward values of the stimuli across participants. The HV preference proportion during the choice test served as the primary outcome variable to evaluate the associations of VL with selective attention in the face and fractal conditions. Details are available in the eMethods in the Supplement.

### Statistical Analysis

The mean HV preference proportions were compared with chance level (0.50) using 2-sided *t* tests in the ASD, DD, and TD groups using the Bonferroni correction for multiple comparisons (.05 ÷ 6 [*P* = .008]). Furthermore, the mean HV preference proportions, mean dwell times, and SRTs were analyzed using a linear mixed-effects model approach. Fixed effects included group (ASD, DD, or TD), condition (faces or fractals), and interactions between group and condition, as well as chronological age and interactions between age and group. Choices of which fixed effects to include and what covariance structure to assume to account for random errors were made using the Akaike information criterion^43^; eTable 3 in the Supplement summarizes this model selection process for each analysis. Final models were fitted using restricted maximum likelihood, with statistical significance evaluated using Kenwood-Roger *df*, and linear contrasts were estimated and tested using least-squares means procedures. Cross-diagnostic associations between task performance and severity of autism symptoms were assessed using Pearson *r* correlation coefficient, partialled for age and nonverbal IQ. Analyses were performed using SAS, version 9.4 (SAS Institute Inc), analytic software.^44^ Details are available in the eMethods in the Supplement.

## Results

### Initial Exclusions

Participant data were excluded from the analysis owing to low quality of eye-tracking data (calibration error >2 degrees), insufficient number of valid trials (<2) during the baseline or choice test phases, or fewer than one-third (<16) valid trials in the training phase of the experiment. Based on these criteria, 7 of 55 children with ASD (13%) were excluded compared with 4 of 35 children with DD (11%) and 2 of 38 children with TD (5%). The exclusion rate did not differ by group (*P* = .49), and the 13 excluded children did not differ statistically significantly from the retained sample in age or autism severity but had lower verbal IQ and nonverbal IQ (descriptive statistics are available in the eMethods in the Supplement). The final sample consisted of 115 preschoolers with ASD (n = 48; mean [SD] age, 38.30 [15.55] months; 37 [77%] boys), DD (n = 31; mean [SD] age, 45.73 [19.49] months; 19 [61%] boys), or TD (n = 36; mean [SD] age, 36.53 [12.39] months; 22 [61%] boys). Table 1 lists other descriptive and test statistics. The 3 groups did not differ statistically significantly in sex distribution. The ASD and TD groups were similar regarding age, but the children in DD group were statistically significantly older than those in the other 2 groups. The ASD and DD groups had comparable verbal IQ and nonverbal IQ, and both groups had lower IQ than the TD group. Predictably, Autism Diagnostic Observation Schedule 2 calibrated severity scores (ADOS-2 CSS) were highest in the ASD group compared with the DD and TD groups. Children with ASD completed a similar number of valid trials as the TD group in both conditions and across all 3 phases (eMethods and eTable 1 in the Supplement). They also completed more trials than the DD group in the training phase of the experiment. The quality of eye-tracking data in the ASD group, as indexed by the mean calibration error, was similar to that in the DD and TD groups (eMethods and eTable 2 in the Supplement).

**Table 1.** Study Sample Characteristics

Variable	ASD (n = 48)	DD (n = 31)	TD (n = 36)	*P* value	Contrasts	*P* value
Male, No. (%)	37 (77)	19 (61)	22 (61)	.27	NA	NA
Age, mean (SD), mo	38.30 (15.55)	45.73 (19.49)	36.53 (12.39)	.05	ASD = TD	.63
					ASD <DD	.04
					DD >TD	.02
ADOS-2 CSS^a^	7.10 (1.81)	2.47 (1.59)	1.18 (0.39)	<.001	ASD >DD	<.001
					ASD >TD	<.001
					DD >TD	.001
Verbal IQ	69.76 (33.17)	80.11 (23.98)	113.29 (17.03)	<.001	ASD = DD	.09
					ASD <TD	<.001
					DD <TD	<.001
Nonverbal IQ	85.39 (20.18)	91.77 (19.15)	114.58 (15.59)	<.001	ASD = DD	.14
					ASD <TD	<.001
					DD <TD	<.001

Abbreviations: ADOS-2 CSS, Autism Diagnostic Observation Schedule 2 calibrated severity scores; ASD, autism spectrum disorder; DD, developmental delay; NA, not applicable; TD, typical development.

^a^ Higher ADOS-2 CSS scores indicate greater severity of autism symptoms.

### Preliminary Analyses

#### Dwell Times at Baseline

To examine whether one class of stimuli had a greater attentional draw over the other at baseline, the mean dwell time was compared across groups and conditions. There was no statistically significant effect of group, and there were statistically significant associations of condition and a group by condition interaction. Within-group comparisons indicated that the differences in the mean dwell time between the face and fractal conditions (face minus fractal) in the ASD group (difference, −6.8 milliseconds; 95% CI, −136.5 to 122.8 milliseconds; *P* = .92) and in the DD group (difference, 132.0 milliseconds; 95% CI, −28.5 to 292.5 milliseconds; *P* = .11) were not statistically significant, suggesting that both groups spent a similar amount of time attending to facial and fractal stimuli (Table 2). However, the mean dwell time was statistically significantly longer in face than fractal conditions in the TD group (difference, 316.2 milliseconds; 95% CI, 168.8-463.6 milliseconds; *P* < .001), suggesting that facial stimuli attracted more attention than fractal stimuli in the TD group.

**Table 2.** Estimated Mean Dwell Time at Stimuli Pairs in the Face and Fractal Conditions in the Autism Spectrum Disorder (ASD), Developmental Delay (DD), and Typical Development (TD) Groups During Baseline

Group	Estimated mean (SE) dwell time, ms		Difference (95% CI), ms	*P* value
	Face condition	Fractal condition		
ASD	1232.0 (55.7)	1238.8 (54.5)	**−**6.8 (−136.5 to 122.8)	.92
DD	1263.3 (69.4)	1131.2 (67.2)	132.1 (**−**28.5 to 292.5)	.11
TD	1467.2 (62.1)	1151.0 (63.8)	316.2 (168.8 to 463.6)	<.001

#### Preference Proportions at Baseline

**Figure 3.**
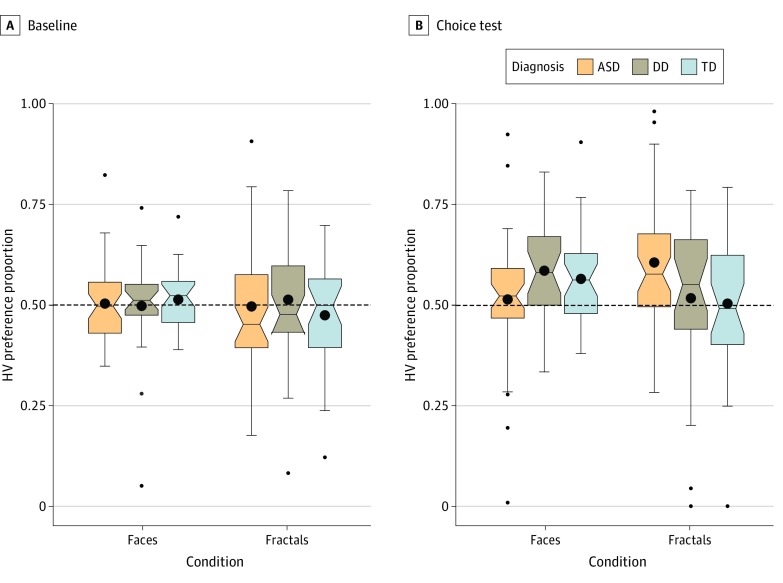
Box Plots Showing the Mean High-Value (HV) Stimulus Preference Proportions During Baseline and During the Choice Test in the Face and Fractal Conditions in the Autism Spectrum Disorder (ASD), Developmental Delay (DD), and Typical Development (TD) Groups A, During baseline, the ASD, DD, and TD groups distributed their attention randomly between the HV and low-value (LV) stimuli in the face and fractal conditions. B, After training, the ASD group showed statistically significant preference for previously reinforced fractals but not faces. However, DD and TD groups showed preference only for previously reinforced faces but not fractals. The dotted line represents chance level performance (0.50). The bottom and top horizontal boundaries of each colored region represent the 25th and 75th percentiles (first and third quartiles Q1 and Q3 and the black dots represent mean values. Vertical lines extend to data values at most 1.5 interquartile ranges (Q3-Q1) above Q3 and below Q1, beyond which outliers are marked as individual data points.

At baseline, HV preference proportions were not statistically significantly different than chance level in any of the groups or conditions (Figure 3). In the face condition, the ASD group had a mean HV preference proportion of 0.50 (95% CI, 0.47-0.53; *P* = .81), and the mean HV preference proportions in the DD and TD groups were 0.50 (95% CI, 0.45-0.55; *P* = .93) and 0.51 (95% CI, 0.49-0.54; *P* = .27), respectively. In the fractal condition, the mean HV preference proportions in the ASD, DD, and TD groups were 0.50 (95% CI, 0.45-0.54; *P* = .88), 0.51 (95% CI, 0.45-0.57; *P* = .66), and 0.48 (95% CI, 0.43-0.52; *P* = .28), respectively.

#### SRT During Training

Subsequently, SRT during training was compared. There was no statistically significant effect of group (*F*_2,109_ = 1.27; *P* = .29) or condition (*F*_1,100_ = 1.10; *P* = .30), but a group by condition interaction (*F*_2,100_ = 5.94; *P* = .004) was statistically significant. In the face condition, the mean SRT of the ASD group was comparable to that of the DD group (ASD minus DD, 21.7 milliseconds; 95% CI, −31.8 to 75.2 milliseconds; *P* = .42) and the TD group (ASD minus TD, 10.0 milliseconds; 95% CI, −40.1 to 60.3 milliseconds; *P* = .69); the DD and TD groups also did not differ statistically significantly (DD minus TD, −11.6 milliseconds; 95% CI, −67.7 to 44.4 milliseconds; *P* = .68). However, in the fractal condition, the ASD group had a shorter SRT than the DD group (ASD minus DD, −61.2 milliseconds; 95% CI, −113.4 to −9.1 milliseconds; *P* = .02) and the TD group (ASD minus TD, −76.5 milliseconds; 95% CI, −127.0 to −25.9 milliseconds; *P* = .003), whereas the DD and TD groups were comparable (DD minus TD, 15.2 milliseconds; 95% CI, −71.0 to 40.6 milliseconds; *P* = .59) (eTable 4 in the Supplement).

Therefore, the 3 groups completed a comparable number of experimental trials, indicating high feasibility of the VL task in participants who were young or developmentally delayed. There were no ASD-specific deficits either in attending to static faces before training or in initiating saccades toward faces during training. Fractals did not appear to have a privileged attentional status compared with faces in participants with ASD before training, but during training those with ASD responded faster to fractal stimuli than the comparison groups, suggesting that dynamic fractal display rapidly acquired a high reward value.

### Associations of VL With Selective Attention

In the face condition during the choice test, the mean HV preference proportion in the ASD group was at chance level (mean, 0.51; 95% CI, 0.46-0.56; *P* = .58), whereas the mean HV preference proportions in the DD group (mean, 0.59; 95% CI, 0.54-0.64; *P* = .001) and the TD group (mean, 0.57; 95% CI, 0.53-0.61; *P* = .002) were statistically significantly above chance level. The pattern was reversed in the fractal condition, where the ASD group showed a mean HV preference proportion greater than chance (mean, 0.61; 95% CI, 0.56-0.65; *P* < .001), but neither the DD group (mean, 0.52; 95% CI, 0.44-0.59; *P* = .64) nor the TD group (mean, 0.50; 95% CI, 0.44-0.57; *P* = .91) showed a similar association (Figure 3). The associations remained statistically significant when the Bonferroni correction for multiple comparisons (.05 ÷ 6 [*P* = .008]) was applied. Therefore, in the ASD group, there was evidence for VL only in the fractal but not in the face condition. Conversely, in the DD and TD groups, learning was apparent only in the face condition but not in the fractal condition.

Linear mixed-effects model analysis on HV preference proportions indicated no statistically significant effects of group (*F*_2,193_ = 0.50; *P* = .61) or condition (*F*_1,193_ = 0.33; *P* = .56), but there was a statistically significant group by condition interaction (*F*_2,193_ = 6.23; *P* = .002). To provide further insight into the interaction, we estimated for each group a differential learning for faces over fractals, defined as the difference between the mean HV preference proportion in the face condition and the mean HV preference proportion in the fractal condition. The differential learning score was statistically significantly lower for the ASD group compared with the DD group (difference between groups, −0.160; 95% CI, −0.267 to −0.054; *P* = .003), and in the ASD group compared with the TD group (difference between groups, −0.153; 95% CI, −0.256 to −0.051; *P* = .005). However, the differential learning score in the DD group and the TD group was comparable (difference between groups, −0.007; 95% CI, −0.120 to 0.106; *P* = .90). The 2 differences identified remained statistically significant when the Bonferroni adjustment for multiple comparisons (.05 ÷ 3 [*P* = .02]) was applied. Within-group comparisons indicated that the incremental learning was statistically significantly lower than zero in the ASD group (estimate, −0.092; 95% CI, −0.159 to −0.025; *P* = .007), but it was not statistically significantly different from zero in the DD group (estimate, 0.068; 95% CI, −0.014 to 0.151; *P* = .11) or the TD group (estimate, 0.062; 95% CI, −0.015 to 0.138; *P* = .12).

### Associations Between VL and Severity of Autism Symptoms

Subsequently, associations were examined between performance on the VL task and autism severity symptoms as measured by the ADOS-2 CSS in the combined ASD, DD, and TD groups, controlling for age and nonverbal IQ. Higher preference for HV faces was associated with lower severity of autism symptoms, (*r* = −0.26; *P* = .01), and higher preference for HV fractals was associated with higher severity of autism symptoms (*r* = 0.22; *P* = .03).

## Discussion

Using a novel VL task, this study found for the first time, to our knowledge, that enhanced value-driven selective attention in the nonsocial domain is associated with ASD in young children compared with DD and TD. This nonsocial learning advantage in the ASD group was accompanied by impaired value-driven selective attention in the social domain. At baseline, preschoolers with ASD looked equally long at face and fractal stimuli pairs; therefore, there was no evidence that nonsocial stimuli were prioritized for processing before training. Most important, enhanced VL in the nonsocial domain and poor VL in the social domain were associated with greater severity of autism symptoms. This study replicates and supports prior work in this area,^37,38,39^ strengthening the evidence regarding disruption in the VL and value-signaling system in ASD. Atypical value-based attention may play a formative role in the emergence of autism symptoms by altering attentional priorities and thus learning opportunities of affected children.

Extant eye-tracking studies in children with ASD do not support the notion of enhanced attention to objects in general,^3,45^ unless the objects fall within an idiosyncratic and highly individualized interest (eg, maps, clocks, and trains).^14,46^ The present study raises the possibility that fractals may map onto largely unrecognized dimensions that turn initially neutral objects into attentionally privileged ones in ASD, although further work is needed to elucidate this phenomenon. Fractals share their self-repeating and scale-invariant properties with patterns in natural scenery^47^ and have a strong aesthetic appeal,^48^ and viewing them may induce relaxation.^49,50^ This study demonstrates in vivo the process through which initially neutral nonsocial stimuli gain privileged status in the attentional system of young children with ASD after a brief exposure. This finding is notable because stimuli deemed to be informative in one context may gain attentional priority when encountered again, despite being irrelevant to the new context.^51^ Furthermore, their priority status may be inappropriately generalized to similar novel stimuli, giving the stimuli unwarranted access to attentional resources.^52^ Overreliance on history and overgeneralization may leave few processing resources for stimuli that are more relevant to adaptive functioning. If present in early life, enhanced VL in the nonsocial domain paired with poor VL in the social domain may have profound implications on the experience-dependent development of neural circuitry that supports the development of attention and cognition. It is not clear whether some classes of nonsocial stimuli gain attentional priority within the first months of life, hijacking the limited attentional resources of infants who later develop ASD, or whether they fill a void left by impaired ability to learn the values of social stimuli later on.

Null results in the face condition cannot be readily explained by inattention or measurement issues because children with ASD completed a comparable number of training and test trials as the TD group, and SRTs during training in the face condition were comparable across groups, implying comparable capacity to orient reflexively toward faces. The results are also not likely owing to difficulty discriminating between the 2 faces because multiple studies^39,53,54,55^ have documented that face discrimination in ASD is intact. This finding leaves the following 2 alternative hypotheses: (1) children with ASD comply with the task and learn the informational value of each face but do not show this knowledge during testing or (2) they comply with the task but have selective impairment in the VL in the social domain. The first hypothesis is informed by the common currency model of value representation,^56,57,58^ which posits that a single subcortical VL circuitry determines the motivational importance of both social and nonsocial stimuli; however, the decision of how to allocate attention is based on the integration of stimulus value with higher-level cognitive information originating from distinct cortical, domain-specific brain areas.^56,57,58^ This framework would suggest that the preschoolers knew which face carried more information but did not prioritize it for processing over the less informative face. The second hypothesis draws on the social valuation–specific model of value representation,^59^ which stipulates that value representation engages distinct cortical and subcortical neural networks in the social and nonsocial domains, with face processing relying on the so-called social brain network. According to this model, value-driven selective attention to faces and fractals may rely on distinct neuronal populations^60^; as such, the observed gaze behaviors in young children with ASD may be driven by dysregulation across both systems that evolved to support learning and signaling of social and nonsocial stimuli values. Both models are plausible given null findings in the face condition, and future neuroimaging studies are needed to directly disambiguate the 2 hypotheses. Such studies would also help to clarify the mechanisms underlying null results in response to fractals in the DD and TD groups.

Although neuroimaging work may help elucidate the neural circuity implicated in atypical value-driven selection in ASD, it will not provide information about how these networks are shaped in ontogenesis. Typically developing newborns orient reflexively to faces, which is mediated by a subcortical mechanism that supports rapid face detection throughout the life span.^61,62,63,64^ Indiscriminate reliance on perceptual salience for guiding attention is neither feasible nor adaptive, and over the course of the first months of life, infants develop a cortically mediated capacity to select stimuli that may not be perceptually salient but are relevant to their adaptation.^65^ Reflexive orienting to faces is preserved in infants, preschoolers, and adults with ASD,^46,66,67^ but infants who later develop ASD fail to make a smooth transition to top-down attentional selection and begin to show limited attention to faces as early as age 6 months^9,10,12^ for reasons that remain to be elucidated.^68,69^ We propose that impaired VL in the social domain may constitute the missing connection between spared reflexive orienting to faces and impaired selective attention to faces in ASD. If social VL deficits emerge shortly after birth, then infants with ASD may accumulate a history of failing to learn the importance of faces in general. Consequently, they may weigh this past failure when encountering novel faces and show limited motivation to engage with them, resulting in a decreased capacity to learn about their informational value. In this context, impaired social VL may constitute a key component in the emergence of atypical social attention in ASD. This hypothesis would be best tested through prospective studies of infants at familial risk for ASD, where the development of VL systems and their association with emerging autistic psychopathology can be evaluated in status nascendi.

### Limitations

This study has some limitations. Although no age associations were found in our sample, further investigation into developmental associations within more narrowly defined epochs will help to assess when the observed deficits first become apparent and identify their long-term effects on the development of social and nonsocial cognition. Replication and extension studies will be necessary to evaluate, among others, the associations of training dose and memory load with VL both in typically developing and developmentally delayed groups. We remain agnostic to the hedonic value of smiling faces vs revolving fractals stimuli because pleasurable sensations derived from the 2 classes of stimuli would be best assessed directly through introspective report, which is not feasible in young and developmentally delayed participants. Future neuroimaging studies may help to disambiguate the learning and hedonic components of task performance.

## Conclusions

To our knowledge, this is the first study to report that atypical selective attention in young children with ASD is associated with a disruption of the processes involved in VL of social and nonsocial information and signaling of value-based attentional priorities. The study shifts emphasis from the investigation of markers of ASD based on descriptive features toward a process-oriented approach.^70^ Investigation into VL provides a highly generative framework for identifying reward-based mechanisms underlying atypical selective attention in children with ASD. Given that structural, neurochemical, and functional facets of reward learning have been established in animal models, this line of inquiry may motivate mechanistic animal model studies on the causal role of value learning dysfunction in ASD.^71^ The greatest separation between ASD, DD, and TD groups was achieved by challenging the cognitive system of children with ASD to learn about the importance of novel social and nonsocial stimuli. This discovery is consequential for the design of novel discriminant and stratification biomarkers in autism.^72^

## Notes

Supplement. **eMethods.** Supplemental Methods **eTable 1.** Mean (SD) Number of Valid Trials Completed by Children in the ASD, DD, and TD Groups During Baseline, Training, and Choice Test Phases in the Face and Fractal Conditions **eTable 2.** Mean (SD) Calibration Accuracy (Degrees of Visual Angle) in the ASD, DD, and TD Groups in the Face and Fractal Conditions **eTable 3.** Comparison of Alternative Models for the Saccadic Reaction Times and Preference Proportions **eTable 4.** Mean (SE) Saccadic Reaction Time (SRT) (in Milliseconds) During the Training Phase in the Face and Fractal Conditions in the ASD, DD, and TD Groups **eReferences.**
