# Supplementary material for: Atypical Value-Driven Selective Attention in Young Children With Autism Spectrum Disorder
Source: JAMA Netw Open. 2020 May 6;3(5):e204928. doi: 10.1001/jamanetworkopen.2020.4928 (PMC7203607; doi:10.1001/jamanetworkopen.2020.4928)
Supplement: Supplement. — eMethods. Supplemental Methods eTable 1. Mean (SD) Number of Valid Trials Completed by Children in the ASD, DD, and TD Groups During Baseline, Training, and Choice Test Phases in the Face and Fractal Conditions eTable 2. Mean (SD) Calibration Accuracy (Degrees of Visual Angle) in the ASD, DD, and TD Groups in the Face and Fractal Conditions eTable 3. Comparison of Alternative Models for the Saccadic Reaction Times and Preference Proportions eTable 4. Mean (SE) Saccadic Reaction Time (SRT) (in Milliseconds) During the Training Phase in the Face and Fractal Conditions in the ASD, DD, and TD Groups eReferences. [file jamanetwopen-3-e204928-s001.pdf]

Supplementary Online Content

Wang Q, Chang J, Chawarska K. Atypical value-driven selective attention in young children with autism spectrum disorder. *JAMA Netw Open*. 2020;3(5):e204928.
doi:10.1001/jamanetworkopen.2020.4928

eMethods. Supplemental Methods

eTable 1. Mean (SD) Number of Valid Trials Completed by Children in the ASD, DD, and TD Groups During Baseline, Training, and Choice Test Phases in the Face and Fractal Conditions

eTable 2. Mean (SD) Calibration Accuracy (Degrees of Visual Angle) in the ASD, DD, and TD Groups in the Face and Fractal Conditions

eTable 3. Comparison of Alternative Models for the Saccadic Reaction Times and Preference Proportions

eTable 4. Mean (SE) Saccadic Reaction Time (SRT) (in Milliseconds) During the Training Phase in the Face and Fractal Conditions in the ASD, DD, and TD Groups

eReferences.

This supplementary material has been provided by the authors to give readers additional information about their work.

© Chawarska K et al. *JAMA Network Open*.

Supplemental Materials

eMETHODS

Participants

Participants. The study was approved by the Human Investigation Committee of the Yale School of Medicine and informed written consent was obtained from all parents prior to testing. The original group of 128 participants included children with ASD (*n* = 55, mean age 38.55 months, *SD* = 15.40), developmental delays and disabilities (DD; *n* = 35, mean age 44.99 months, *SD* = 18.99), and typically developing controls (TD; *n* = 38, mean age 36.43 months, *SD* = 12.07) recruited between March 3, 2017 and June 13, 2018. Participants with ASD and DD were recruited from consecutive referrals to the Yale Toddler Developmental Disabilities Clinic by parents or health care providers. TD participants were recruited through online advertisements and community outreach. Diagnosis was assigned by a team of expert clinicians based on a review of the child's medical and developmental history, as well as direct assessments of autism symptom severity and cognitive functioning.

Cognitive skills were evaluated using either the Mullen Scales of Early Learning (MSEL) (90/128 or 70% of participants), the Bayley Scales of Infant and Toddler Development – 3rd Edition (5/128 or 4% of participants), or the Differential Ability Scales-II Early Years(DAS-II) (33/128 or 26% of participants) depending on age and ability level.[1] Autism severity was quantified using the Autism Diagnostic Observation Schedule-2 (ADOS-2). Ten out of 128 children (9 in the TD group and 1 in the DD group) did not receive the ADOS-2 assessment due to time constraints. The ADOS-2 Toddler Module was administered to 43/118 or 36% of

© Chawarska K et al. *JAMA Network Open*.

participants, while Modules 1, 2, and 3 were administered to 15/118 (13%), 42/118 (36%), and 18/118 (15%) of the sample, respectively. The ASD group consisted of children who received a clinical best estimate (CBE) diagnosis of ASD and had an ADOS-2 calibrated severity score (CSS) above the ASD diagnostic 4-point cut-off. The DD group included children with specific or global developmental delays (*n* = 29), as well as ADHD, anxiety, social difficulties, or behavioral problem symptoms (*n* = 6). Children in the DD group underwent evaluation of their social-communicative skills using the ADOS-2 and an ASD diagnosis was ruled out by expert clinicians. Children in the TD group had a CBE classification of normative development, ADOS-2 CSS of 3 or below, and verbal and nonverbal IQ scores above 85.

Participant data were excluded from the analysis due to low quality of eye-tracking data (calibration error > 2 degrees), insufficient number of valid trials (< 2) during *Baseline* or *Choice Test* phases, or because the participants completed fewer than 1/3 (< 16) of valid trials in the *Training* phase of the experiment. Based on these criteria, 7/55 (12.71%) ASD participants were excluded, compared to 4/53 (11.43%) DD and 2/38 (5.26%) TD participants (*P* = .49). The children excluded from the analysis did not differ significantly from the retained sample in age [*M* = 37.06 (SD = 11.89) months *versus M* = 39.69 (*SD* = 16.15) months; *P* = .58) and autism symptom severity [*M* = 5.27 (*SD* = 3.63) *versus M* = 4.25 (*SD* = 3.06); *P* = .303], but had lower verbal IQ [*M* = 65.98 (*SD* = 35.38) *versus M* = 86.18 (*SD* = 32.40); *P* = .044] and nonverbal IQ scores [*M* = 79.28 (*SD* = 27.58) *versus M* = 96.16 (*SD* = 22.37); *P* = .016]. After initial exclusions, the final sample consisted of 48 out of 55 (87%) children with ASD, 31 out of 35 (89%) children with DD, and 36 out of 38 (95%) TD children (see **Table 1** for sample characteristics).

Stimuli

A single pair of faces and a single pair of fractals were presented as stimuli, and all participants were exposed to these same four stimuli. To generate these stimuli, 12 fractal videos were produced using a generic algorithm-based fractal generation software[2] and 12 videos of female happy facial expressions were drawn from the BU-4DFE 3D Dynamic Facial Database.[3] The opening frames of the videos were selected as the static stimuli. To ensure that the two classes of stimuli were equivalently perceptually salient, the perceptual salience of each stimulus in all 24 videos was rated on 10-point scales by 17 adults through crowdsourcing[4] via Qualtrics. For the face stimuli, the magnitude of each emotional expression (neutral and happy) was also rated. Each stimulus was rated twice, first as a static still-frame representing the first frame of the video and again as a dynamic display. Means of all ratings were calculated across all stimuli. Subsequently, one pair of faces and one pair of fractals with saliency rating within a half point of the overall mean were selected to be used in this study. Stimuli sets were also standardized regarding their luminance, contrast density, texture similarity, size, color, intensity, and positioning.[5] See **Figure 1** for a representation of the static versions of the stimuli.

Procedure

Each *Baseline* phase consisted of four free-viewing trials per participant to help examine any pre-existing attentional biases among the images prior to *Training*. During each *Baseline* phase trial, the HV stimulus (i.e., the stimulus that was later reinforced during *Training*) was presented simultaneously with the LV stimulus (i.e., the stimulus that was not reinforced during *Training*) for 4000 ms in two out of four possible locations, selected at random. The stimuli

© Chawarska K et al. *JAMA Network Open*.

were static images, consisting of the first frame of the videos later presented during the *Training* phase. Each stimulus subtended approximately 9 degrees of visual angle and was presented with an eccentricity of approximately 4 degrees from the central fixation point, consisting of an expanding colorful circle subtending approximately 1 degree of visual angle. Each of the four trials began with a presentation of a central fixation point for 600 ms. The offset of the central fixation point coincided with the presentation onset of the pair of stimuli (fractals or faces) lasting for 4000 ms. A gray screen was presented during each inter-trial interval (ITI), lasting randomly between 400 ms and 800 ms.

The *Training* phase consisted of 48 trials during which the HV and LV stimuli were presented 24 times in a random order and stimuli were never presented in the same location for more than two consecutive trials. Each trial started the presentation of a central fixation point (multi-color pulsating circle) for 600 ms, followed by the presentation of either the HV or the LV stimulus in one of four randomly selected locations for 2000 ms. There was a 300 ms overlap between the presentation of the central fixation point and the stimulus to diminish the likelihood that participants would look away from the screen prior to the onset of the peripheral stimulus. If a child fixated a HV stimulus, it underwent a visual transformation: the fractal would revolve and the face would smile. However, when the child failed to fixate on the HV stimulus, it remained static. If a child fixated a LV stimulus, the stimulus remained static. The reward value of each stimulus within a pair was randomized across subjects, so that in approximately half of the subjects, face 1 (or fractal 1) was reinforced during *Training* (HV stimulus), while face 2 (or fractal 2) was not reinforced (LV stimulus).

The free-viewing *Choice Test* phase consisted of six trials with the same structure as the *Baseline* trials, whereby HV and LV stimuli were presented simultaneously for 4000 ms in two out of four possible location selected at random. No reinforcement was given for looking at HV stimuli. To minimize the effect of memory load, choice tests were administered immediately after each participant completed the training phase. Eye tracking data were collected throughout the experiment.

Analysis of valid trials counts

During *Baseline* and *Choice Test*, a trial was considered valid if the participant had a total mean dwell time on either one or both of the stimuli greater than 100 ms and the calibration accuracy was below 2 degrees of visual angle. During *Training*, a trial was considered valid it the child shifted gaze toward the peripheral stimulus. Means and standard deviations for the counts of valid trials across all groups and phases of the experiment are shown in **eTable 1**.

In the *Baseline* phase, linear mixed effects model analysis on the number of valid trials, with group, condition, group x condition, and age as fixed effects revealed no effects of group, $F(2,112) = 1.26$, $P = .289$, condition, $F(1,91) = 0.40$, $P = .530$, or interaction between group and condition, $F(2,91) = .05$, $P = .951$. The contribution of chronological age to the model was not significant ($P = .15$). Out of 4 possible trials, participants completed an average of 3.84 (SD = 0.46) trials. During the *Training* phase, LMM analysis indicated a significant effect of group, $F(2,112) = 3.23$, $P = .043$, but no effect of condition, $F(1,91) = 1.32$, $P = .253$, or interaction between group and condition, $F(2,91) = 1.50$, $P = .228$. The effect of age was not significant ($P = .376$). Post-hoc between-group comparisons indicated that children in the ASD group

completed more *Training* trials than children in the DD group (*P* = .039) and a comparable number of trials to the TD group (*P* = 1.00); DD and TD groups did not differ (*P* = .280). Finally, during *Choice Test*, LMM analysis indicated no effect of group, *F*(2,112) = 0.88, *P* = .419, or condition, *F*(1,91) = 3.92, *P* = .051, but a significant group x condition interaction, *F*(2,91) = 3.33, *P* = .040. The effect of age was not significant (*P* = .243). Post-hoc between-group comparisons across the Face condition revealed no significant differences between ASD and DD groups (*P* = .218) or TD (*P* = .336) groups, or between DD and TD groups (*P* = 1.00). Similarly, there were no group differences in the Fractal condition between ASD and DD (*P* = .168) or TD (*P* = 1.00) groups, or between DD and TD groups (*P* = .187). Within-group comparisons indicated that ASD and TD groups had a comparable number of valid trials during *Choice Test* in the Fractal and Face conditions (*P* = .589 and *P* = .293, respectively), but the DD group contributed fewer trials in the Fractal than the Face condition (*P* = .007).

eTable 1. Mean (SD) number of valid trials completed by children in the ASD, DD, and TD groups during *Baseline*, *Training*, and *Choice Test* phases in the Face and Fractal conditions.

Phase	ASD		DD		TD	
	Face	Fractal	Face	Fractal	Face	Fractal
N	42	44	27	29	34	32
Baseline	3.90 (0.37)	3.84 (0.48)	3.74 (0.59)	3.72 (0.53)	3.91 (0.38)	3.87 (0.42)
Training	37.19 (7.70)	38.18 (5.61)	35.41 (7.26)	34.17 (8.51)	37.76 (5.59)	35.53 (6.00)
Choice Test	5.05 (1.17)	5.18 (1.11)	5.48 (0.75)	4.72 (1.16)	5.41 (0.89)	5.19 (0.82)

© Chawarska K et al. *JAMA Network Open*.

Accuracy of eye tracking data

After invalid trials were excluded, calibration accuracy was computed for the three groups and two conditions. Linear mixed effects model analysis on calibration accuracy, with group, condition, age, and age x group interaction as fixed effects, indicated no significant effects of group (*P* = .34), condition (*P* = .61), age (*P* = .57), group x condition interaction (*P*=.77), or age x group interaction (*P* = .69). Thus, there were no significant differences between groups in calibration accuracy in either the Face or the Fractal conditions (**eTable 2**).

eTable 2. Mean (standard deviation) calibration accuracy (degrees of visual angle) in the ASD, DD, and TD group in the Face and Fractal conditions.

Condition	ASD<br>M(SD)	DD<br>M(SD)	TD<br>M(SD)	p-value
Face	0.69 (0.32)	0.79 (0.42)	0.64 (0.29)	.23
Fractal	0.63 (0.35)	0.76 (0.36)	0.66 (0.30)	.25

Outcome Measures

Dependent measures. For individual i, phase p (baseline, training, or choice test), condition c (face or fractal), and value v (HV or LV, for high or low value, respectively), we defined $t_i(p, c, v)$ to be the average dwell time for individual i in phase p for condition c and value v. The average was taken over all valid trials, that is, it was the sum of dwell times divided by the number of valid trials.

Preliminary Analyses. As a preliminary analysis, to investigate inherent differences in attention to the two categories of stimuli across diagnostic groups before training, we

© Chawarska K et al. *JAMA Network Open*.

calculated a baseline mean dwell time for each condition c in each individual i by averaging the mean dwell times for the two stimuli (HV and LV) from that condition, that is,

$$b_i(c) = \frac{1}{2}\left(t_i(\text{baseline}, c, \text{HV}) + t_i(\text{baseline}, c, \text{LV})\right)$$

In this way, for each individual i we obtained $b_i(\text{face})$ and $b_i(\text{fractal})$, that is, average dwell time for faces and for fractals in the baseline phase. We also evaluated differences across diagnostic groups in the ability to generate reactive saccades from the central fixation point toward the target face or fractal stimulus presented peripherally during *Training.* With this aim, we calculated two saccadic reaction time (SRT) indices for each participant, one for the Faces condition and one for the Fractals condition, by averaging the time intervals between the onset of the peripheral stimulus and the time when the participant first fixated on the stimulus.

Primary Outcome Variable. The primary outcome variable indexing HV stimulus preference was the HV preference proportion, defined as

$$r_i(p, c) = \frac{t_i(p, c, \text{HV})}{t_i(p, c, \text{HV}) + t_i(p, c, \text{LV})}$$

for individual i, phase p, and condition c. This proportion was evaluated during the *Baseline* phase to check for pre-existing attentional biases based on perceptual salience and during the *Choice Test* phase to examine the effects of value training.

Statistical Analysis

eTable 3. Comparison of alternative models for the saccadic reaction times and preference proportions. All models include main effects and interactions for diagnosis and condition. For age effects, "age|dx" includes an interaction between age and dx, and for covariance structures, the "|dx" notation indicates that a different covariance matrix is estimated for each diagnostic group. Akaike Information Criterion (AIC) values for maximum likelihood (ML) and restricted maximum likelihood (REML) fits are shown. For AIC, the smaller the better, so AIC favors the models in the top row of each table. Some values are omitted because it is not meaningful to compare REML AIC values for models having different fixed effects.

(a) Models for dwell time analysis.

Age effects	Covariance structure	AIC (ML)	AIC (REML)
none	compound symmetry	3041.2	2969.1
age	compound symmetry	3041.7	
age|dx	compound symmetry	3044.9	
none	spherical	3050.4	2977.9
none	diagonal	3051.8	2979.3
none	unstructured	3042.6	2970.5
none	compound symmetry|dx	3045.3	2973.3
none	unstructured|dx	3048.2	2976.2

The best model for analysis of dwell time at Baseline according to AIC included fixed effects of diagnosis, condition, and their interaction as fixed effects, but not age, with a covariance matrix having compound symmetry structure.

(b) Models for saccadic reaction time analysis.

Age effects	Covariance structure	AIC (ML)	AIC (REML)
none	compound symmetry	-331.1	-305.9
age	compound symmetry	-330.4	
age|dx	compound symmetry	-330.2	
none	Spherical	-316.3	-274.2
none	Diagonal	-315.5	-290.9
none	unstructured	-330.6	-305.4
none	compound symmetry|dx	-323.6	-298.4
none	unstructured|dx	-322.1	-296.8

The best model for the SRT analysis according to AIC included fixed effects of diagnosis, condition, and their interaction as fixed effects, but not age, with a covariance matrix having compound symmetry structure.

(c) Models for HV preference proportion analysis.

Age effects	Covariance structure	ML AIC	REML AIC
none	diagonal	-172.8	-152.3
age	diagonal	-172.5	
age|dx	diagonal	-172.6	
none	spherical	-169.1	-148.7
none	compound symmetry	-167.8	-147.4
none	unstructured	-171.5	-150.9
none	diagonal|dx	-171.3	-150.5
none	unstructured|dx	-169.0	-148.1

The best model for the HV preference proportions analysis according to AIC included fixed effects of diagnosis, condition, and their interaction, but not age, and the best covariance matrix structure was diagonal, that is, with preference proportions in the Face and Fractal conditions being uncorrelated with different variances.

Preliminary Analyses

eTable 4. Mean (SE) saccadic reaction time (SRT) (in milliseconds) during the *Training* phase in the Face and Fractal conditions in the ASD, DD, and TD groups.

	ASD	DD	TD	Contrasts	Difference (95% CI)
Face (ms)	422.4 (17.0)	400.7 (21.2)	412.3 (19.0)	ASD = DD, *P* = .42	21.71 (-31.8 to 75.2)
				ASD = TD, *P* = .69	10.08 (-40.1 to 60.3)
				TD = DD, *P* = .68	11.63 (-67.7 to 44.4)
Fractal (ms)	378.8 (16.6)	440.1 (20.5)	455.3 (19.5)	ASD < DD, *P* = .02	-61.24 (-113.4 to -9.1)
				ASD < TD, *P* = .003	-76.46 (-127.0 to -25.9)
				TD = DD, *P* = .59	-15.22 (-71.0 to 40.6)

© Chawarska K et al. *JAMA Network Open*.

References

1. APA. *American Psychiatric Association: Diagnostic and Statistical Manual of Mental Disorders: DSM 5.* 5th ed. ed. Washington, DC: Author; 2013.
2. Draves S. The electric sheep screen-saver: A case study in aesthetic evolution. *Applications on Evolutionary Computing.* 2005:458–467.
3. Zhang X, Yin L, Cohn J, et al. BP4D-Spontaneous: A high resolution spontaneous 3D dynamic facial expression database. *Image and Vision Computing.* 2014;32(special issue of the Best of FG13): 692-670.
4. Redi JA, Hoßfeld T, Korshunov P, Mazza F, Povoa I, Keimel C. Crowdsourcing-based multimedia subjective evaluations: a case study on image recognizability and aesthetic appeal. *Proceedings of the 2nd ACM international workshop on Crowdsourcing for multimedia* 2013: 29–34.
5. Itti L. Quantifying the contribution of low-level saliency to human eye movements in dynamic scenes. *Visual Cognition.* 2005;12(6):1093-1123.
6. Casey B, Oliveri ME, Insel T. A neurodevelopmental perspective on the research domain criteria (RDoC) framework. *Biological psychiatry.* 2014;76(5):350-353.

© Chawarska K et al. *JAMA Network Open*.
